# Mechanisms of tunneling nanotube-based propagation of neurodegenerative disease proteins

**DOI:** 10.3389/fnmol.2022.957067

**Published:** 2022-07-15

**Authors:** Sarita Lagalwar

**Affiliations:** Neuroscience Program, Skidmore College, Saratoga Springs, NY, United States

**Keywords:** tunneling nanotubes, synuclein, tau, ataxin-1, prions, autophagy

## Abstract

Tunneling nanotubes (TNTs), intercellular connections enriched with F-actin, were first identified as a viable means of cellular communication and organelle transport in animal cells at the early part of this century. Within the last 10 years, these microscopic and highly dynamic protrusions have been implicated in neurodegenerative disease propagation and pathogenesis. A host of aggregation-prone protein inclusions, including those containing alpha-synuclein, tau, prions and others, hijack this communication mechanism in both neurons and astrocytes. The exact cellular mechanisms underlying TNT-based propagation remain largely unknown, however, common practices can be identified. First, selective expression of the aggregation-prone form of proteins increases TNT density; next, endo-lysosomal pathways appear to support the loading and unloading of protein onto the TNT; and finally, TNT assembly results in the spontaneous formation of aggregation-prone protein inclusions in “acceptor” cells, indicating that TNTs are involved in not only the transport of inclusions but also in the seeding of new inclusions in naïve cells. These observations have implications for the spreading of neurodegenerative disease in the central nervous system and the consequent progression of symptoms. Here, I will summarize the empirical evidence of TNT-based aggregation-prone protein propagation to date, and propose an inclusive model of aggregate inclusion propagation along TNTs.

## Introduction

Tunneling nanotubes (TNTs) are long, thin, cell-to-cell connections between mammalian cells, including neurons, that have been identified in recent years as a mechanism of cellular communication. Several thorough review articles have been written which define TNTs and detail their characteristics (Baluska et al., 2004; Gerdes et al., 2007; Gerdes and Carvalho, 2008; Gurke et al., 2008; Rustom, 2009). In brief, TNTs are membrane-bound extensions of cells lined with f-actin and free of microtubules. They have been shown to shuttle endosomes, lysosomes, mitochondria and other materials including proteins and nucleotides between cells. They share several structural and functional similarities with plant plasmodesmata including the use of the actin-myosin transport system to shuttle their cargo between cells.

Unlike plasmodesmata, *in vivo* detection of TNTs have been lacking, with the strongest *in vivo* support of TNTs being localization within human glioblastomas implanted into mouse brains (Osswald et al., 2015). *In vitro* support is mounting however, with TNT data being generated in both cancer and neurodegenerative disease research. TNTs have been identified in a number of cell lines as well as in a growing number of primary cells. With respect to the neurodegenerative disease field, questions still exist. Are TNTs used for the bi-directional transport of organelles and other materials? Is the purpose of TNTs in fact cellular communication? If so, do foreign viruses and pathogenic proteins simply hijack the machinery for their propagation needs? Or is the purpose of TNTs for support of disease cells by healthy cells to accept pathogenic proteins and damaged organelles and provide disease cells with functional organelles? Finally, is TNT formation responsible for the spreading of neurodegenerative disease within and across brain regions? In this mini-review, I will examine the TNT literature on propagation of aggregation-prone proteins to date and to provide a model of aggregate inclusion propagation by TNTs based on that evidence.

## Aggregation-prone protein expression enhances tunneling nanotubes formation

Tunneling nanotubes formation has been identified in a variety of cultured cell lines as a means of managing the assault of aggregation-prone protein expression, both through overexpression and through internalization. Costanzo et al. (2013) observed that within 24 h of culturing, TNTs appeared in CNS-derived catecholaminergic CAD cells over-expressing GFP-480, or GFP-480 fused to the N-terminus of the hungtingtin (htt) protein containing either 17Q polyglutamines (aggregation-resistant) or 68Q polyglutamines (aggregation-prone). At 48 h in culture, TNT formation increased by 20% in the aggregation-prone cells while no increase is seen in the aggregation-resistant or control GFP-480 cells, and corresponds to an increase in aggregate formation of GFP-480-68Q (20%) compared to GFP-480-17Q (5%). The authors identified transfer of aggregates into mCherry-containing acceptor cells by co-culture and confirmed aggregate movement across TNTs as the mechanism by which transfer occurred. Aggregate transfer of GFP-480-68Q was re-produced in primary cerebellar granule cell cultures (Costanzo et al., 2013).

Zhu et al. (2015) identified 20% increased TNT formation in CAD cells overexpressing GFP-PrP^C^ compared to control cells overexpressing non-fused GFP. Interestingly, there was no corresponding increase in the transfer of Vybrant™ DiD-labeled vesicles in the PrP^C^ cells indicating an “uncoupling” of transfer from TNT formation. The authors suggest that perhaps what they identified as TNTs were not fully developed, or perhaps TNT formation may be an intermediate stage which requires more active mechanisms of molecular motor expression, energy production and more to carry it out in full. A third explanation is that overexpression of mutated, and therefore aggregation-prone, GFP-PrP^C^ (GFP-PrP^SC^) would induce greater rate of transfer compared to either overexpressed GFP-PrP^C^ or overexpressed GFP. The same study found that chronically-infected CAD (ScCAD) cells did show a 20% increase in TNT formation compared to CAD cells which corresponded to an increase in DiD-labeled vesicle transfer (28% in ScCAD vs. 16% in CAD) (Zhu et al., 2015).

**FIGURE 1:**
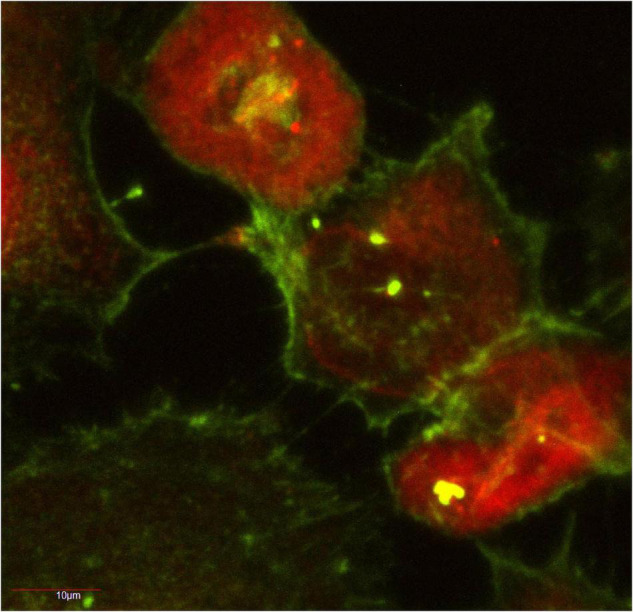
Human medulloblastoma Daoy cells stably transfected with RFP-ATXN1(82) at 72 h in culture. The actin-positive TNT network is shown in green. RFP-ATXN1(82Q) aggregates are seen in several of the TNTs. A portion of this figure was published in Huang et al. (2022), CC BY 4.0.

Our lab demonstrated that stable over-expression of the RFP-fused aggregation-prone ataxin-1 protein [RFP-ATXN1(82Q)] in human medulloblastoma-derived Daoy cells as well as transient over-expression of GFP-fused aggregation-prone ataxin-1 protein [GFP-ATXN1(85Q)] in mouse neuroblastoma neuro2A cells led to TNT formation and subsequent aggregate transfer across TNTs within 72 h (Figure 1; Huang et al., 2022). We did not find extensive TNT formation in cells expressing aggregation-resistant forms of ataxin-1 [RFP-ATN1(82Q-A776), RFP-ATXN1(30Q), GFP-ATXN1(32Q)], nor did we see the extracellular presence of these proteins.

Abounit et al. (2016b) prepared *in vitro* fibrils of human α-synuclein (Abounit et al., 2016a) and tau (hTau-Alexa-555). Infection of CAD cells with α-synuclein fibrils led to internalization of fibrils and a subsequent 25% increase in TNT formation compared to non-infected CAD cells. Using a donor-acceptor cell model, the authors confirmed cell-to-cell TNT transfer of fibrils. Infection and internalization of hTau-Alexa-555 increased TNT numbers in HeLa (> 20%) and CAD cells (30%) compared to non-infected control cells. Using iPSC-derived astrocytes, Rostami et al. (2017) identified increased TNT formation in a non-neuronal and non-tumorigenic cell culture model infected with α-synuclein oligomers. In their model, non-infected cells maintained approximately 10 TNTs over a 6-day period in culture, while in infected cells, the number of TNTs grew from just under 20–25 in that same time period. The actin polymerization inhibitor latrunculin B inhibited the number of TNTs present by 75%.

In contrast to the studies outlined above, Dieriks et al. (2017) did not detect any difference in the number of TNTs formed in isolated brain pericytes or SH-SY5Y neuroblastoma cells when stably expressed with α-synuclein-A53T-EGFP, α-synuclein-WT-EGFP, α-synuclein-WT-mCherry or non-fused mCherry as a control. While they did see transfer of α-synuclein, there was greater detection of plasma membrane transfer without respect to the presence of α-synuclein indicating that TNT-mediated transfer is part of a broader, more general mechanism of cellular communication. When that form of communication is involved in the transport of benign entities such as RNA or vesicles, it has remained seemingly undetected.

## Endolysosomal involvement in tunneling nanotubes formation

### Proteasomal and lysosomal dysfunction

Proteasomal and lysosomal dysfunction accompanies expression of aggregation-prone proteins, and as a consequence, ubiquitin-proteasomal and autophagic fail-safe mechanisms are disrupted. Chastagner et al. (2020) overexpressed, *via* lentiviral infection, tau RD-YFP (P301L-mutated microtubule binding domain of tau protein) in SHSY-5Y cells. Subsets of aggregated tau-RD complexes co-localized with ubiquitin and a subset co-localized with p62, an adaptor of autophagy which recognizes autophagic cargo. However, neither proteasomal degradation nor autophagy cleared the aggregates, and moreover, aggregates were found to be distinct from membrane-bound endosomes, lysosomes, autophagosomes, Golgi or mitochondria. Rather, they were produced or accumulated directly in the cytoplasm. Attempts to manipulate tau aggregation clearance by application of bafilomycin A1, an inhibitor of late-stage autophagy *via* inhibition of autophagosome-lysosome fusion, and application of Bortezomide, a proteasome enzyme complex inhibitor, had little effect. Similarly, our study (Huang et al., 2022) found that induction of autophagy with 500 nM rapamycin or arrest of autophagy by 60 μM chloroquine redistributed diffuse RFP-ATXN1[82Q] into aggregates but did not clear the aggregates. Notably, induction of autophagy clears aggregation-resistant and diffuse RFP-ATXN1[30Q] protein, while arrest of autophagy induced the formation of RFP-ATXN1[30Q] into large aggregates. RFP-ATXN1[82Q] aggregates did not co-localize with ubiquitin; upon proteasomal inhibition by 100 nM lactacystin, a subset of aggregates became ubiquitinated but did not clear.

Infection of iPSC-derived astrocytes by α-synuclein oligomers led to co-localization between the lysosomal marker LAMP-1 and oligomers 3 days post-exposure. At 6 days post-exposure, co-localization was no longer evident, but the oligomers remained and were stored in the *trans* Golgi network, leading to endoplasmic reticulum stress, mitochondrial fragmentation and autophagic dysfunction. Attempts at mitophagy were made by astrocytes, however, pathological mitochondria remained. Interestingly, oligomer-containing astrocytes responded to the dysfunction by building out TNTs to healthy acceptor cells to which damaged mitochondria were shuttled out. In turn, the control cells provided healthy mitochondria to the oligomer-containing astrocytes. Imaging and counts of mitotracker-positive mitochondria confirmed that control astrocytes delivered more healthy mitochondria to α-synuclein oligomer-infected acceptor cells (50%) than healthy control acceptor cells (30%).

### Membrane organelle recycling

While tau RD-YFP aggregates did not reside in vesicle membranes (Chastagner et al., 2020), Prp^SC^ puncta along ScCAD cell-TNTs, following guanidium thiocyanate denaturation, co-localized with the endosomal marker EEA1 (28%), lysosomal marker LAMP1 (40%) and the endocytic recycling compartment protein Vamp3 (45%). Percentages are reflective of independent experiments (Zhu et al., 2015). Their results suggest membrane surface Prp^SC^ is continuously recycled. While attempts are made by the cell to degrade the proteins *via* autophagy, dysfunction of autophagic mechanisms requires the cell to shuttle out autophagic cargo *via* TNTs.

Abounit et al. (2016a) found that α-synuclein fibril infection of donor cells co-localized with EEA1 (< 20%), Vamp3 (> 20%) and LAMP1 (50%). Fibrils identified along TNTs were largely LAMP1-positive. Following a 24-h infection period, analysis of acceptor cells indicated that a majority of fibrils co-localized with LAMP1 (< 30%), while smaller amounts of fibrils co-localized with EEA1 (3%) and Vamp3 (< 30%). The study was repeated using the lysosome marker LysoTracker, and found that 80% of lysosomal vesicles in donor cells which transferred to acceptor cells contained α-synuclein fibrils. The results strongly suggested that fibril-filled lysosomes were undergoing direct cell-to-cell transfer.

## Transport and seeding

The fate of aggregation-prone proteins shuttled from diseased cells to healthy cells would logically be expected to end in proteasomal or lysosomal degradation by healthy, active and functioning ubiquitin-proteasome systems and autophagic processes. While we cannot discount the likely possibility of targeted degradation, evidence exists that in (perhaps, *some*) instances, the load of aggregation-prone proteins shuttled to acceptor cells overwhelms those processes, and instead, aggregation-prone proteins seed aggregation-resistant proteins to aggregate.

Chastagner et al. (2020) found that fibrils of K18 tau (synthetic PHF core tau) infected into acceptor CAD cells spread to neighboring cells where they seeded aggregates of full-length Tau 1N4R P301S fused to YFP (FLTau). FLTau aggregation was dependent on, and partially co-localized with, K18 tau. Importantly, using a biosensor system combined with an Incu-Cyte-automated incubator microscope, the authors recorded images every 30 min for 3 days. RD-YFP aggregates from K18 tau-challenged cells propagated over several generations. In addition, Huang et al. (2022) found that aggregation-prone RFP-ATXN1[82Q] caused aggregation-resistant proteins GFP-ATXN1[32Q] and GFP-ATXN1[85Q-S776A] to form small and medium-sized aggregates which co-localized with RFP-ATXN1[82Q]. RFP-ATXN1[82Q] caused aggregation-prone GFP-ATXN1[85Q] to form large-sized aggregates which co-localized with RFP-ATXN1[82Q], a double-seeding effect. Finally, GFP-ATXN1[85Q] caused aggregation-resistant RFP-ATXN1[82Q-S776A] to form small and medium aggregates which co-localized with GFP-ATXN1[85Q].

## Discussion

Plasmodesmata allow for intercellular communication between plant cells. The relatively recent finding that mammalian neuronal and non-neuronal cells induce formation of structures similar to plasmodesmata, TNTs, in response to expression or inoculation of aggregation-prone proteins introduces the question of what role TNTs play in neurodegenerative disease. One option is to target TNTs pharmacologically as a means of slowing seeding. Dilsizoglu Senol et al. (2019) discerned the efficacy of the cyanobacterial macrolide tolytoxin, which disrupts actin dynamics through inhibition of actin polymerization and induction of fragmentation of f-actin. Without alterations to microtubules or intermediate filaments, Dilsizoglu Senol et al. (2019) found that 3 and 15 nM tolytoxin dissolved in methanol reduced the number of TNTs in SW13 and SHSY-5Y cells, respectively. 15 nM tolytoxin reduced α-synuclein fibril transfer (Control- 40%, methanol- 42%, tolytoxin- 20%) and the number of α-synuclein puncta in the cell. However, healthy mitochondrial transfer was reduced as well (control-50%, methanol- 40%, tolytoxin- 10%). Therapeutics targeting TNT formation may therefore not be safe or effective given their likelihood to interfere with endogenous protective mechanisms when taken early in the disease process and their predicted ineffectiveness at inhibiting seeding when taken late in the disease process.

**FIGURE 2:**
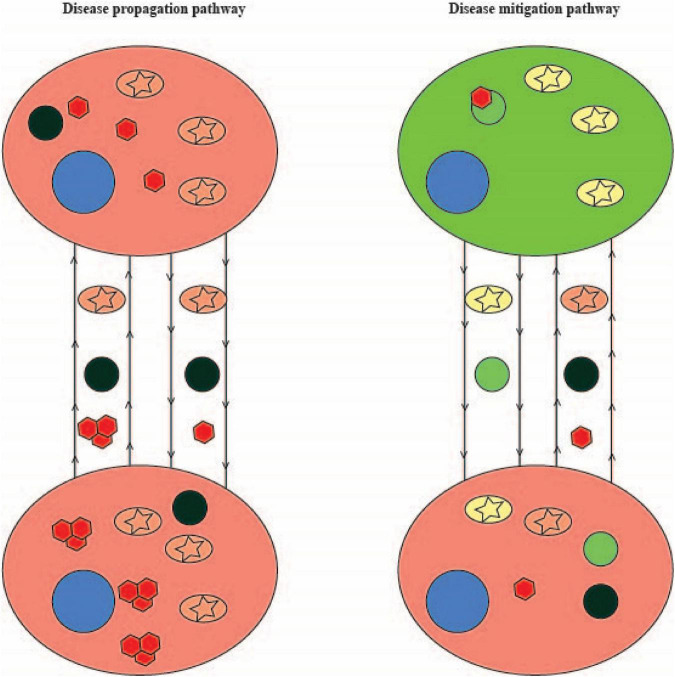
A model of the role that TNTs may play in neurodegeneration. On the left are two disease cells (red circles) undergoing neurodegeneration and connected by TNTs. TNTs shuttle dysfunctional mitochondria (pink circles with stars), dysfunctional lysosomes (dark green circles), and aggregation-prone proteins (red hexagons) between cells. The accumulation of aggregation-prone protein in disease cells leads to seeding and enlargement of aggregates (depicted by three adjacent red hexagons). On the right is a healthy cell (large light green circle) connected to a disease cell (red circle) by TNTs. The disease cell shuttles dysfunctional mitochondria, dysfunctional lysosomes and aggregation-prone proteins to the healthy cell. The healthy cell degrades the aggregation-prone proteins in lysosomes and shuttles healthy lysosomes (light green small circles) and mitochondria (yellow circles with stars) to the disease cell. As a result, the disease cell is able to manage its load of aggregation-prone protein. Nuclei are depicted with blue circles and arrowheads reflect the direction of transport.

The studies in particular by Abounit et al. (2016a) and Rostami et al. clearly identified organelle transfer along TNTs and Rostami et al. further identified coupled transfer by which inclusions and mitochondria travel in opposing directions. Taken together, their work as well as the other studies illustrated here suggest a model by which amplification of TNT formation as a protective mechanism allows diseased or vulnerable cells to export their inclusions and in exchange import healthy mitochondria and lysosomes. However, Chastagner et al. (2020) and Huang et al. (2022) demonstrate seeding of non-aggregation prone proteins by aggregation-prone proteins following transfer. How do we reconcile the two seemingly disparate functions of TNTs? Do they form in order to slow the disease process *via* coupled transfer with healthy cells or do they form to enhance the propagation of aggregation-prone proteins thereby speeding up the disease process? Or, is the natural limit by which healthy organelle import can occur (either due to weakening of healthy cells due to coupled transfer or loss of healthy cells due to disease propagation) the point at which seeding occurs? Figure 2 summarizes a potential model of the role that TNTs play in the neurodegenerative disease process. In this model, coupled transfer between a healthy and disease cell allows the healthy cell to provide functional working organelles (particularly mitochondria and lysosomes) to disease cells. In turn, the disease cell shuttles off damaged organelles and aggregation-prone proteins to the healthy cell for degradation. Through this pathway disease is mitigated. In contrast, as the disease process progresses and healthy cells become diseased or die off, TNTs formed between disease cells results in damaged organelles being shuttled back and forth. Shuttling of aggregation-prone protein leads to seeding (potentially even double-seeding events), exacerbating the disease process in seeded cells. As a result, disease propagates through this pathway. While this model is based on the *in vitro* studies outlined in this mini-review, further work in *in vivo* models are needed in order to better establish the extent that TNTs are an integral part of neurodegenerative disease propagation.

## Author contributions

The author confirms being the sole contributor of this work and has approved it for publication.

## Conflict of interest

The author declares that the research was conducted in the absence of any commercial or financial relationships that could be construed as a potential conflict of interest.

## Publisher’s note

All claims expressed in this article are solely those of the authors and do not necessarily represent those of their affiliated organizations, or those of the publisher, the editors and the reviewers. Any product that may be evaluated in this article, or claim that may be made by its manufacturer, is not guaranteed or endorsed by the publisher.

## References

[B1] Abounit S. Bousset L. Loria F. Zhu S. de Chaumont F. Pieri L. (2016a). Tunneling nanotubes spread fibrillar alpha-synuclein by intercellular trafficking of lysosomes. *EMBO J.* 35 2120–2138. 10.15252/embj.201593411 10.15252/embj.201593411 27550960 PMC5048354

[B2] Abounit S. Wu J. W. Duff K. Victoria G. S. Zurzolo C. (2016b). Tunneling nanotubes: a possible highway in the spreading of tau and other prion-like proteins in neurodegenerative diseases. *Prion* 10 344–351. 10.1080/19336896.2016.1223003 27715442 PMC5105909

[B3] Baluska F. Hlavacka A. Volkmann D. Menzel D. (2004). Getting connected: actin-based cell-to-cell channels in plants and animals. *Trends Cell. Biol.* 14 404–408. 10.1016/j.tcb.2004.07.001 15308205

[B4] Chastagner P. Loria F. Vargas J. Y. Tois J. I Diamond M. Okafo G. (2020). Fate and propagation of endogenously formed Tau aggregates in neuronal cells. *EMBO Mol. Med.* 12:e12025. 10.15252/emmm.202012025 33179866 PMC7721367

[B5] Costanzo M. Abounit S. Marzo L. Danckaert A. Chamoun Z. Roux P. (2013). Transfer of polyglutamine aggregates in neuronal cells occurs in tunneling nanotubes. *J. Cell. Sci.* 126(Pt 16), 3678–3685. 10.1242/jcs.126086 23781027

[B6] Dieriks B. V. Park T. I. Fourie C. Faull R. L. Dragunow M. Curtis M. A. (2017). alpha-synuclein transfer through tunneling nanotubes occurs in SH-SY5Y cells and primary brain pericytes from Parkinson’s disease patients. *Sci. Rep.* 7:42984. 10.1038/srep42984 28230073 PMC5322400

[B7] Dilsizoglu Senol A. Pepe A. Grudina C. (2019). Effect of tolytoxin on tunneling nanotube formation and function. *Sci. Rep.* 9:5741. 10.1038/s41598-019-42161-6 30952909 PMC6450976

[B8] Gerdes H. H. Carvalho R. N. (2008). Intercellular transfer mediated by tunneling nanotubes. *Curr. Opin. Cell. Biol.* 20 470–475. 18456488 10.1016/j.ceb.2008.03.005

[B9] Gerdes H. H. Bukoreshtliev N. V. Barroso J. F. (2007). Tunneling nanotubes: a new route for the exchange of components between animal cells. *FEBS Lett.* 581 2194–2201. 10.1016/j.febslet.2007.03.071 10.1016/j.febslet.2007.03.071 17433307

[B10] Gurke S. Barroso J. F. Gerdes H. H. (2008). The art of cellular communication: tunneling nanotubes bridge the divide. *Histochem. Cell. Biol.* 129 539–550. 10.1007/s00418-008-0412-0 18386044 PMC2323029

[B11] Huang H. Toker N. Burr E. Okoro J. Moog M. Hearing C. (2022). Intercellular propagation and aggregate seeding of mutant ataxin-1. *J. Mol. Neurosci.* 72 708–718. 10.1007/s12031-021-01944-1 34826062 PMC8986690

[B12] Osswald M. Jung E. Sahm F. (2015). Brain tumour cells interconnect to a functional and resistant network. *Nature* 528 93–98. 26536111 10.1038/nature16071

[B13] Rostami J. Holmqvist S. Lindström V. Sigvardson J. Westermark G. T. Ingelsson M. (2017). Human astrocytes transfer aggregated alpha-synuclein via tunneling nanotubes. *J. Neurosci.* 37 11835–11853. 29089438 10.1523/JNEUROSCI.0983-17.2017 PMC5719970

[B14] Rustom A. (2009). Hen or egg?: some thoughts on tunneling nanotubes. *Ann. N. Y. Acad. Sci.* 1178 129–136. 19845633 10.1111/j.1749-6632.2009.04997.x

[B15] Zhu S. Victoria G. S. Marzo L. Ghosh R. Zurzolo C. (2015). Prion aggregates transfer through tunneling nanotubes in endocytic vesicles. *Prion* 9 125–135. 25996400 10.1080/19336896.2015.1025189 PMC4601206

